# Post-Irradiation Sinus Mucosa Disease in Nasopharyngeal Carcinoma Patients Treated with Intensity-Modulated Proton Therapy

**DOI:** 10.3390/cancers14010225

**Published:** 2022-01-04

**Authors:** Pei-Wen Wu, Chien-Chia Huang, Yun-Shien Lee, Yung-Chih Chou, Kang-Hsing Fan, Chien-Yu Lin, Bing-Shen Huang, Shih-Wei Yang, Chi-Che Huang, Po-Hung Chang, Ta-Jen Lee, Joseph Tung-Chieh Chang

**Affiliations:** 1 Division of Rhinology, Department of Otolaryngology, Chang Gung Memorial Hospital and Chang Gung University, Taoyuan 333, Taiwan; a9665@cgmh.org.tw (P.-W.W.); agar@cgmh.org.tw (C.-C.H.); hcc3110@cgmh.org.tw (C.-C.H.); bc1766@gmail.com (P.-H.C.); 2 Department of Otolaryngology–Head and Neck Surgery, Chang Gung Memorial Hospital and Chang Gung University, Keelung 204, Taiwan; sweeyang@gmail.com; 3 Graduate Institute of Clinical Medical Sciences, College of Medicine, Chang Gung University, Taoyuan 333, Taiwan; 4 Genomic Medicine Research Core Laboratory, Chang Gung Memorial Hospital, Taoyuan 333, Taiwan; bojack@mail.mcu.edu.tw; 5 Department of Biotechnology, Ming Chuan University, Taoyuan 333, Taiwan; 6 Department of Radiation Oncology, Chang Gung Memorial Hospital and Chang Gung University, Taoyuan 333, Taiwan; russellhome88@gmail.com (Y.-C.C.); khs.fan@gmail.com (K.-H.F.); qqvirus1022@gmail.com (C.-Y.L.); beanson.tw@gmail.com (B.-S.H.); 7 Department of Radiation Oncology, New Taipei Municipal Tucheng Hospital, New Taipei City 236, Taiwan

**Keywords:** nasopharyngeal carcinoma, post-irradiation sinusitis, sinus mucosa disease, proton therapy

## Abstract

**Simple Summary:**

Chronic rhinosinusitis (CRS) is a common treatment complication in patients with nasopharyngeal carcinoma (NPC) after radiotherapy. In this study, we aimed to investigate the incidence and severity of CRS in NPC patients who underwent intensity-modulated proton therapy (IMPT) by evaluating the sinus mucosa change in imaging studies, and we compared these patients with those who underwent volume-modulated arc therapy (VMAT). This was a retrospective case–control study in which 53 and 54 patients were treated with IMPT and VMAT, respectively. We noted that patients in the IMPT group had a significantly lower incidence and decreased severity of sinus mucosa abnormality than those with VMAT. Better and faster recovery of sinonasal function after radiotherapy in the IMPT group was also observed. IMPT techniques deposit the bulk of their radiation doses in highly confined areas, allowing lower exposure of non-target organs during irradiation, which results in more sinonasal mucosa being retained.

**Abstract:**

In the past decade, patients with nasopharyngeal cancer (NPC) have been deemed candidates for proton radiotherapy, due to the large and comprehensive target volumes and the necessity for the retention of the surrounding healthy tissues. In this study, we aimed to compare the incidence and severity of post-irradiation sinusitis by detecting sinus mucosa diseases (SMDs) via the magnetic resonance imaging (MRI) of patients with NPC after intensity-modulated proton therapy (IMPT) and volume-modulated arc therapy (VMAT). A total of 53 patients in the IMPT group and 54 patients in the VMAT group were enrolled in this study. There were significantly lower endoscopic scores and Lund–Mackay staging scores determined from MRI scans in the IMPT group during different follow-up periods. For the most vulnerable sinuses, the incidence and severity of SMD were the highest during the third post-radiotherapy month in both groups. These decreased steadily, and there was no significant increase in the incidence and severity of SMD during the second post-radiotherapy year in the IMPT group. Our data show that NPC patients with IMPT have a significantly lower incidence and decreased severity of SMD than those with VMAT. A better and faster recovery of sinonasal function after radiotherapy in the IMPT group was also observed.

## 1. Introduction

Nasopharyngeal carcinoma (NPC) is endemic in a few areas of Asia because of the complex interaction between viral, genetic, and environmental risk factors [1,2]. In Taiwan, the overall age-standardized incidence rate of NPC among adults was reported to exceed 7 per 100,000 population, peaking at ages 40–50. A male predominance, by 3:1, was also observed [3]. The public health issues and socioeconomic burden in relation to this disease deserve further attention and consideration.

Due to the anatomical location of the nasopharynx and the highly radiosensitive cancer cells, radiation therapy is the standard treatment for NPC. Evolution of radiation therapy in the past two decades, from two-dimensional planning to intensity-modulated radiation therapy (IMRT) or volume-modulated arc therapy (VMAT) has improved target volume coverage and decreased the radiation doses obtained by the surrounding normal tissue [4]. Consequently, improved cure rates and a reduction in long-term toxicity, as a result of the integration of adjuvant systemic therapy and advancements in radiation therapy delivery, were observed [5]. However, the incidence of post-irradiation sinus mucosa disease remains high [6,7,8].

In the treatment of NPC, the radiation volume covers the nasopharynx, posterior nasal cavity, skull base, sphenoid sinus, posterior parts of the ethmoid and maxillary sinus, oropharynx, and upper neck [9]. Although the paranasal sinuses are usually not considered to be radiosensitive tissues according to the literature regarding tolerance doses for organs in the head and neck region [10], increasing evidence observed the irreversible damage to the epithelial lining by irradiation [11,12,13,14,15,16]. Nasal cytology analysis demonstrated a higher percentage of neutrophilic inflammation, squamous cell metaplasia, and mucous cell metaplasia in treated patients [13,14]. Radiation therapy causes sinonasal epithelial cells to become degenerative and more prone to desquamation and ciliary dysfunction [16]. These adverse effects of treating NPC significantly increase the susceptibility of the oropharynx to infections, resulting in overlaid infections in the sinuses [17]. In a single institutional study of irradiated patients with NPC, chronic rhinosinusitis (CRS) was found in up to 73% of patients and was the most common treatment complication [18]. Notably, CRS has negative impacts on quality of life and results in a great physical, psychological, and economic burden.

The role of proton beam therapy in treating head and neck cancer has been rapidly expanding. Treatment with protons differs from the photon radiation used in IMRT or VMAT in a basic and important mode: protons deposit the bulk of the radiation dose in a highly confined zone, resulting in a minimal-to-zero dose reaching surrounding normal tissues located beyond the specified depth [19]. A matched retrospective case–control study [20] of patients treated with three-dimensional conformal proton therapy (3DCPT) and IMRT observed a lower incidence of gastrostomy tube dependence (20% for 3DCPT vs. 65% for IMRT) attributed to improved oral cavity sparing, as was seen in another retrospective combined cohort study [21] of NPC, nasal cavity, and paranasal sinus cancers. The change in sinonasal mucosa in NPC patients treated with proton beam therapy has not been evaluated. In addition, intensity-modulated proton therapy (IMPT) has better radiation dose conformality than 3DCPT does. Thus, this study aimed to evaluate the incidence and severity of sinus mucosa disease (SMD) in patients with NPC after IMPT, by using the Lund–Mackay (L–M) staging system in serial magnetic resonance imaging (MRI) studies, and to compare these patients with those treated with VMAT.

## 2. Materials and Methods

### 2.1. Patients

**Figure 1:**
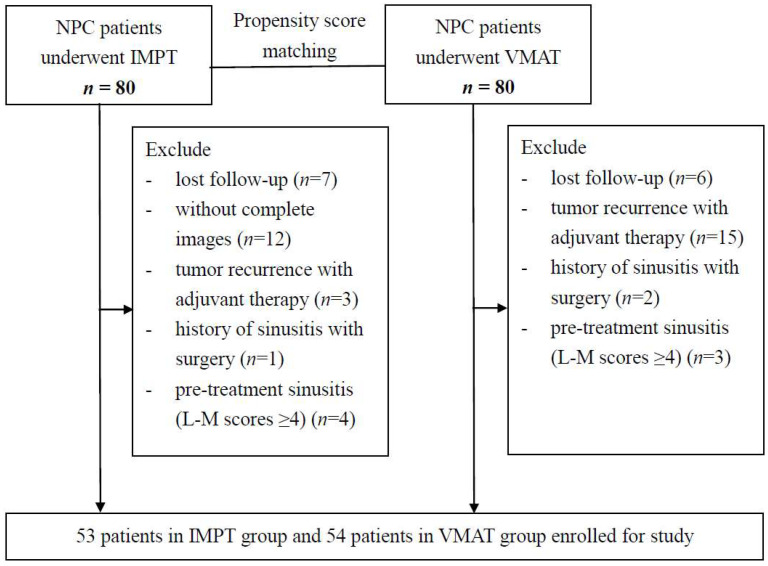
Flow chart of patient selection process.

The study population consisted of the patients in the previous matched-comparison NPC study treated with IMPT or VMAT that were treated between 2014 and 2018 [22]. Among these patients, those with incomplete follow-up imaging studies, tumor recurrence with adjuvant therapy, follow-ups of less than 2 years, history of functional endoscopic sinus surgery for CRS, and pre-treatment sinusitis (L–M scores ≥ 4) were excluded (Figure 1). In total, 53 patients in the IMPT group and 54 patients in the VMAT group were enrolled for analysis. All the patients received a standard protocol comprising a physical examination, nasopharyngoscopy, MRI of the head and neck region, and a whole-body tumor scan. The 8th edition of the American Joint Committee on Cancer (AJCC) staging system for NPC was applied for disease staging.

### 2.2. Radiotherapy Protocol

The used radiotherapy was described in detail in a previous study [22]. The radiotherapy prescriptions were based on the prospective clinical trial NRG-HN001 [23]. A high-risk clinical tumor volume (CTV6996) was defined as gross disease plus a 3–5 mm margin, and the prescribed dose was 69.96 Gy for VMAT, or 69.96 Gy (relative biological effectiveness (RBE)) for IMPT, given in 2.12 Gy or Gy (RBE) fractions. An RBE value of 1.1 was assumed for protons. The high-risk subclinical region (CTV5940) was 59.4 Gy in 33 fractions (1.8 Gy/fraction), in some cases. A low-risk clinical tumor volume (CTV5412) was 54.12 Gy in 33 fractions (1.64 Gy/fraction) for N0 and low neck (level IV and V). Another 3 or 5 mm planning target volume (PTV) was added to the CTV volumes, depending on the image-guided radiotherapy for patients treated with VMAT. Another 5–10 mm was added for the setup error, motion, and range uncertainty in IMPT planning at the beginning of proton use, but it was reduced to around 5 mm in recent years after robust optimization and evaluation became routinely used. For robust optimization in the IMPT planning, robust worst-case optimization was used for CTV coverage without PTV expansion. The Eclipse planning system (version 13.7; Varian Medical Systems, Palo Alto, CA, USA) with the pencil beam line scanning system was used for IMPT and VMAT planning. The full-field IMPT plans used three different beam angles simultaneously. There were two different compositions of the three angles: left and right anterior oblique and a single posterior beam, or left and right posterior oblique and single rear beam. We used the left and right posterior oblique angles for patients with too many metal fillings in their teeth because the CT artifacts increased the range uncertainty. The planning system optimized all the spots from all the fields simultaneously.

Most patients received concurrent chemotherapy, and no patients had adjuvant chemotherapy. The patients with stage I disease were treated with radiotherapy alone. The mainly concurrent chemotherapy PUL regimen was 50 mg/m^2^ of cisplatin (P) on Day 1, followed by tegafur plus uracil (U; 300 mg/m^2^/day) plus leucovorin (L; 60 mg/day) daily for 14 days [23]. A few patients with advanced-stage cancer had induction chemotherapy. The induction chemotherapy regimen typically consisted of platinum with docetaxel or gemcitabine.

### 2.3. Protocol of MRI

MRI evaluation was performed at 1.5 Tesla units using a standard head coli. A spin-echo technique was used. MRI was performed on all the patients before and after a gadolinium–DTPA injection. Images were acquired in the sagittal, axial, and coronal planes. The section thicknesses were 5 mm with a 2.5 mm intersection gap in the axial plane and 4 mm with a 1 mm gap in the sagittal and coronal planes.

### 2.4. Post-Treatment Follow-Up

All the irradiated patients were scheduled for clinic visits at 1-month intervals in the first three months after treatment and every 3 months thereafter. Structured interviews for clinical manifestations, nasopharyngoscopy for the detection of local recurrence, and physical examinations of the neck were carried out during every visit. A follow-up MRI of the head and neck region was performed 3 months after the completion of radiotherapy and then 6–12 months yearly, or when clinically indicated.

### 2.5. Evaluation of Paranasal Sinus

The L–M staging system is traditionally used for the evaluation of the severity of CRS, and it designates a score for the ostiomeatal complex (OMC) and each sinus group (maxillary, anterior ethmoid, posterior ethmoid, sphenoid, and frontal sinuses) according to computed tomography (CT) scan findings [24]. The sinuses are scored between 0 and 2 (0, no abnormality; 1, partial opacification; 2, total opacification). MRI, which was used for the pre-treatment staging and follow-up of head and neck structures in the current study, possesses the advantage of excellent tissue contrast and is superior to CT for evaluating the extent of tumors [25]. We modified the L–M staging system by omitting scoring for OMCs because the bony structure could not be precisely evaluated via the MRI modality. The status of the sinus mucosa was assessed by documenting mucosal changes on the MRI, and the scores of the individual sinus were obtained before radiotherapy and 3 months, 1 year, and 2 years after treatment. All the L–M scores were assigned with consensus by two otolaryngologists (C.-C.H. and P.-W.W.).

### 2.6. Evaluation of Nasopharynx

Nasopharyngoscopy was performed during each clinical visit after treatment. A modified Lund–Kennedy (L–K) endoscopic scoring system [26], which retains the L–K subscores of discharge and crusting, was used to evaluate the localized inflammation of the irradiated nasopharyngeal mucosa. The discharge of the nasopharynx was scored between 0 and 2 (0, absent; 1, clear discharge; 2, purulent discharge), and the crusting was scored between 0 and 3 (0, absent; 1, crust on less than 50% of the nasopharynx; 2, crust on over 50% of the nasopharynx; 3, crust extending beyond the nasopharynx). All endoscopic scores were assigned with consensus by two otolaryngologists (C.-C.H. and P.-W.W.).

### 2.7. Statistical Methods

Statistical analysis was performed using MATLAB 2015b, The MathWorks, Natick. Patients with IMPT and VMAT were divided into two groups. Univariate analysis of categorical variables was performed using Fisher’s exact test, while the Mann–Whitney U test was used for comparison of continuous variables. The differences in the incidence and severity of sinus mucosa abnormality at successive time periods in each treatment group were analyzed using the Mann–Whitney *U* test. A *p*-value of < 0.05 was considered to be statistically significant.

## 3. Results

### 3.1. Patients and Tumor Characteristics

In total, 53 patients undergoing IMPT and 54 patients undergoing VMAT were enrolled in this study. There was no significant difference in age, sex, pre-treatment L–M score, or tumor stages between the two study groups (Table 1). All patients were followed up for 2 years or more.

### 3.2. Post-Irradiation Sinus Mucosa Abnormality and Localized Nasopharyngeal Inflammation

Both sides of the paranasal sinuses (including maxillary, anterior/posterior ethmoid, sphenoid, and frontal sinuses) in each patient were independently analyzed. Hence, there were 106 sinuses in the IMPT group and 108 sinuses in the VMAT group. The L–M scores of individual sinuses on the MRI and the endoscopic scores were obtained 3 months, 1 year, and 2 years after treatment. Patients with VMAT had significantly higher mean L–M scores and endoscopic scores than those with IMPT (*p* < 0.001). This difference was observed in all the different follow-up periods (Table 2). In addition, both scores in each group showed a decreasing trend over time.

### 3.3. Abnormal Rates of SMD before and after Radiation Therapy

The incidences of SMD (score one or more) in the maxillary, anterior/posterior ethmoid, sphenoid, and frontal sinuses in each group before radiotherapy, and three months, one year, and two years after radiotherapy, are summarized in Table 3. The sinuses could be generally categorized into high-risk (maxillary and anterior ethmoid sinuses), medium-risk (posterior ethmoid and sphenoid sinuses), and low-risk (frontal sinus) groups according to the vulnerability to treatment toxicity [8]. The incidence of SMD in medium- and low-risk sinuses in the IMPT group showed no significant change before and after treatment. The adverse effect of proton beam therapy on the maxillary sinus and anterior ethmoid sinuses resolved during the first and second post-radiotherapy year, respectively, when compared to pre-treatment. On the other hand, patients with VMAT showed significantly high rates of sinus mucosa abnormality in the maxillary, anterior, and posterior ethmoid sinuses. The adverse effect of photon beam therapy persisted in the second post-radiotherapy year.

### 3.4. Chronological Changes in Severity of SMD during Different Follow-Up Periods

**Figure 2:**
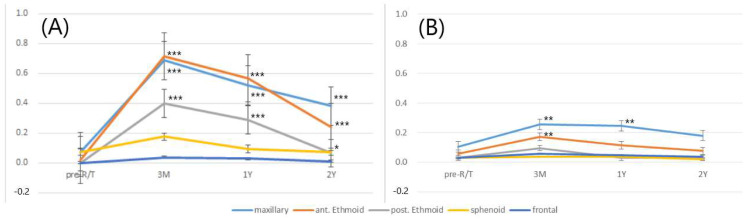
Mean values of the Lund–Mackay scores for individual sinuses in patients with nasopharyngeal carcinoma before and after volume-modulated arc therapy (**A**) and intensity-modulated proton therapy (**B**) in different follow-up periods. (*** *p* < 0.001, ** *p* < 0.01, and * *p* < 0.05).

The changes in the mean L–M scores for each sinus in different follow-up periods represent the changes in the severity of SMD, as shown in Figure 2. In the VMAT group, for maxillary, anterior ethmoid, and posterior ethmoid sinuses, a significant increase in the score from the third month to the second year after radiotherapy, when compared to pre-treatment, was observed. By contrast, in the IMPT group, significant changes were solely observed from the third month to the first year after radiotherapy for the maxillary sinus, and the third month after radiotherapy for the anterior ethmoid sinus. In both groups, for sphenoid and frontal sinuses, there was no remarkable change in the mean L–M scores during the pretreatment and different post-radiotherapy follow-up periods.

## 4. Discussion

Adverse effects of radiation therapy are common in patients with NPC because the exposure of non-target normal tissue during the irradiation of the head and neck areas is inevitable. Lou et al. noted that sinonasal epithelial changes, such as ciliary loss, ciliary dysmorphism, and inter- and intra-cellular vacuolation, could be observed 23 years after irradiation in NPC patients [12]. The ciliary dysfunction predisposes the patient to recurrent upper respiratory tract infections, resulting in refractory CRS. Patients may suffer from purulent anterior and posterior nasal drainage, nasal obstruction, facial congestion, facial pain/pressure/fullness, hyposmia, and fever, which all result in a significantly impaired quality of life [27]. Patients are usually educated to use nasal irrigation for the maintenance of nasal hygiene and to assist in the recovery of sinonasal mucociliary clearing in clinical practice. However, a study conducted by Ayoub N et al. suggested that nearly 50% of these patients receiving conservative treatment reported it to be ineffective and finally needed to receive endoscopic sinus surgery [28]. The adage “prevention is better than cure” is as important today as it has always been. The de-intensification and personalization of radiation therapy to limit toxicity are of renovated importance in treating NPC patients. Most patients will be cured, but they bear the consequences of treatment toxicity for decades [19].

This study is the first to investigate the incidence and severity of sinonasal mucosa toxicity after IMPT and to make comparisons with those following VMAT. We retrospectively reviewed SMD on MRI scans to evaluate the status of the sinonasal mucosa. As Bassiouny et al. suggested, the radiologic extension of the SMDs reflected the reduction in the ciliary area and in the ciliary count studied via scanning electron microscopy [29]. Herein, we used MRI instead of CT as an investigation tool to differentiate between the SMD and tumor involvement. Modified L–M staging scores, omitting the score for OMCs, were applied to represent the severity of sinonasal mucosa toxicity after treatment. In general, compared to patients who underwent VMAT, patients treated with IMPT had lower mean L–M scores at 3 months, 1 year, and 2 years after treatment. In addition, there was less discharge and crusting on irradiated nasopharyngeal mucosa in the IMPT group in all the follow-up periods. These may correlate with the irreplaceable physical characteristics of the Bragg peak, where most of the proton beam radiation dose is deposited across a narrow range of depth, reducing the unavoidable irradiation of surrounding normal tissues [19]. The improved sinonasal retention during IMPT may result in better mucociliary clearance. This retention preserves the essential protective functions of the airway in protecting patients from recurrent upper respiratory airway infections and CRS.

Huang et al. [6] and Hsin et al. [8] found that nearly one-third (32.1% and 31.4%, respectively) of NPC patients had SMDs before treatment. The prevalence rate was similar to the rates reported regarding the incidental sinus abnormality seen on MRI scans in the normal population [30,31,32]. Ashraf et al. also reported that the mean prorated L–M score (excluding scoring for OMCs) was 4.26 (95% CI, 3.55 to 4.97) in patients undergoing CT of the paranasal sinus region for non-sinusitis causes [33]. Thus, in the current study, for precisely evaluating the radiotherapy toxicity exerted on the sinus mucosa, patients with pre-treatment L–M scores ≥ four were excluded. As a result, in the VMAT group, there was an approximately 9-fold and 38-fold incidence of SMD in the acute stage (third post-radiotherapy month) for the maxillary and anterior ethmoid sinuses, respectively, much higher than the two-fold increase reported by Hsin et al. [8]. By contrast, in the IMPT group, only two- and three-fold increases in the incidence of SMD were observed in the acute stage for the maxillary and anterior ethmoid sinuses, respectively. These significant differences indicate that IMPT is better than VMAT in preserving sinonasal mucosa function, especially in the acute stage after radiotherapy.

Regarding the different sinus groups, previous studies showed that both the maxillary and anterior ethmoid sinuses are the most vulnerable areas for treatment toxicity after VMAT for NPC [6,7,8]. Exposure to radiation is not the only factor related to the pathogenesis of post-irradiation sinusitis. It is likely that these post-irradiation changes also share the common etiology of usual rhinosinusitis. This suggests that a wealth of bony and mucosal structures in the relatively narrow OMC are vulnerable to obstruction and, hence, critical in the generation of rhinosinusitis [34]. Our data showed a significant change in SMD incidence in the VMAT group for the maxillary, anterior ethmoid, and posterior ethmoid sinuses, during all different follow-up periods. The severity of the sinus mucosal change, quantified with the L–M staging scores, was also noteworthy.

By contrast, in the IMPT group, a significant increase in SMD incidence was only seen in the acute stage (third post-radiotherapy month) for both the maxillary and anterior ethmoid sinuses. The acute mucosal changes were not observed in the first post-radiotherapy year, except for the maxillary sinus. Either the incidence or severity of SMD decreased during the follow-up period. They became no longer statistically significant in the second post-radiotherapy year for all the paranasal sinuses. The differences between the two study groups indicated that proton beam therapy, especially IMPT, exerts more negligible toxicities on the sinus in the long term than VMAT does.

This study has several limitations that warrant consideration. First, it was a retrospective study. We used L–M scores obtained with the MRI modality to represent the severity of sinus mucosa damage after radiotherapy. However, information regarding the subjective symptoms and quality-of-life questionnaires related to the sinonasal domain was lacking. Studies have shown that the L–M staging system for CRS does not always correlate well with clinical parameters, likely due to its coarse scale [35,36]. Further studies evaluating the correlation between post-radiotherapy symptoms, patient-based questionnaires quantitating the impact on quality of life, and L–M staging scores are essential. Second, most of the patients in this study had nasopharyngoscopy follow-up focusing on the nasopharynx, not the sinonasal area. We could not clarify the relationship between endoscopic and radiologic findings, which is essential in diagnosing sinusitis. Third, several factors affect sinonasal mucociliary clearance. A prospective study with a long-term observation of these findings and correlations is required. In addition to radiation characteristics, nasal atopy, the efficacy and frequency of nasal irrigation, smoking habits, and environmental exposure are all involved in the pathogenesis of CRS. Further investigation should be considered. Finally, this study had a retrospective case–control design. A large-scale prospective study is needed to gain further information.

## 5. Conclusions

Our study investigated the incidence and severity of SMD in NPC patients who received VMAT and IMPT, the first case–control comparison to date. With improvements in the application of IMPT in treating head and neck cancer, proton techniques have proved able to facilitate a reduction in the off-target exposure of multiple organs to the radiation. Improved sinonasal mucosa retention was expected. We noted that NPC patients receiving IMPT had a significantly lower incidence and decreased severity of SMD than those receiving VMAT did. Better and faster recovery of sinonasal function after radiotherapy in the IMPT group was also observed. The clinical benefits of proton beam therapy for patients with NPC are becoming increasingly apparent.

## Figures and Tables

**Table 1:** Patients and tumor characteristics.

		IMPT (*n* = 53) No. of Patients (%) *	VMAT (*n* = 54) No. of Patients (%) *	*p*-Value
**Age (years)**		48.28 ± 13.38	50.31 ± 10.36	0.38
**Gender**	Male	39 (73.6)	45 (83.3)	0.82
	Female	14 (26.4)	9 (16.6)	
**Pre-RT L–M scores**		0.49 ± 0.93	0.33 ± 0.73	0.33
**Tumor classification**	T1	22 (41.5)	25 (46.3)	0.86
	T2	8 (15.1)	6 (11.1)	
	T3	13 (24.5)	11 (20.4)	
	T4	10 (18.9)	12 (22.2)	
**Nodal classification**	N0	10 (18.9)	10 (18.5)	0.56
	N1	29 (54.7)	24 (44.4)	
	N2	5 (9.4)	10 (18.5)	
	N3	9 (17.0)	10 (18.5)	
**Stage group**	I	6 (11.3)	5 (29.3)	0.92
	II	17 (32.1)	16 (29.6)	
	III	10 (18.9)	13 (24.1)	
	IV	20 (37.7)	20 (37.0)	

Data are represented as mean ± standard deviation. Abbreviations: IMPT, intensity-modulated proton therapy; VMAT, volume-modulated arc therapy; RT, radiotherapy; L–M, Lund–Mackay. * Amounts may not sum to 100% because of rounding.

**Table 2:** Comparison of severity of post-irradiation sinus mucosa abnormality and localized nasopharyngeal inflammation in nasopharyngeal carcinoma patients treated with intensity-modulated proton therapy and volume-modulated arc therapy.

		IMPT	VMAT	*p*-Value
3 m post-RT	L–M score	0.72 ± 1.61	4.02 ± 2.73	<0.001
	Endoscopic score	1.42 ± 0.82	3.02 ± 0.81	<0.001
1 y post-RT	L–M score	0.70 ± 1.48	2.98 ± 2.52	<0.001
	Endoscopic score	1.25 ± 0.96	3.00 ± 0.95	<0.001
2 y post-RT	L–M score	0.68 ± 1.58	1.54 ± 1.94	<0.001
	Endoscopic score	1.08 ± 1.03	2.41 ± 1.11	<0.001

Data are represented as mean ± standard deviation. Abbreviations: RT, radiotherapy; L–M, Lund–Mackay; IMPT, intensity-modulated proton therapy; VMAT, volume-modulated arc therapy.

**Table 3:** Incidences of mucosal abnormalities in 106 sinuses of patients treated with intensity-modulated proton therapy and 108 sinuses of patients treated with volume-modulated arc therapy.

	Maxillary		Ant. Ethmoid		Post. Ethmoid		Sphenoid		Frontal	
	IMPT	VMAT	IMPT	VMAT	IMPT	VMAT	IMPT	VMAT	IMPT	VMAT
Pre-RT	11(10.3)	7(6.5)	6(5.7)	2(1.9)	3(2.8)	0(0)	3(2.8)	6(5.7)	2(1.9)	0(0)
3 m post-RT	24(22.6) **	66(61.1) ***	18(17.0) **	77(71.3) ***	9(8.4)	34(31.5) ***	3(2.8)	14(13.2)	3(2.8)	3(2.8)
1 y post-RT	23(21.7) **	53(49.1) ***	12(11.3)	61(56.5) ***	2(1.9)	28(25.9) ***	3(2.8)	9(8.5)	3(2.8)	3(2.8)
2 y post-RT	17(16.0)	39(36.1) ***	8(7.5)	26(24.1) ***	2(1.9)	6(5.6) *	1(0.9)	7(6.6)	2(1.9)	1(1.0)

No. of patients (%). * *p* < 0.05 when compared chronologically to pre-RT data. ** *p* < 0.01 when compared chronologically to pre-RT data. *** *p* < 0.001 when compared chronologically to pre-RT data. Abbreviations: RT, radiotherapy; IMPT, intensity-modulated proton therapy; VMAT, volume-modulated arc therapy.

## Data Availability

The data presented in this study are available in this article.
